# Family Resilience and Children’s Subjective Well-Being: A Two-Wave Study

**DOI:** 10.3390/children11040442

**Published:** 2024-04-07

**Authors:** Andreja Brajša-Žganec, Marija Džida, Maja Kućar

**Affiliations:** Ivo Pilar Institute of Social Sciences, 10 000 Zagreb, Croatia; marija.dzida@pilar.hr (M.D.); mkucar@pilar.hr (M.K.)

**Keywords:** family resilience, subjective well-being, preadolescence, life satisfaction, positive affect, negative affect

## Abstract

According to the Theory of Change, the resilience of the family unit plays a crucial role in shaping the developmental trajectory of children. Families exhibiting higher levels of family resilience are typically characterized by transparent and effective communication, optimistic outlooks on adversity, adept problem-solving skills, strong spiritual beliefs, and effective management of social and financial resources. While existing research has indicated that parental and familial characteristics can predict diverse outcomes for children, investigations concerning the association between family resilience and children’s subjective well-being remains limited. Therefore, this study aims to examine whether different dimensions of family resilience can predict changes in children’s subjective well-being, tested one year later. The sample includes 762 child-mother-father triads (intact families). Children aged 9–13 years (48% boys, age = 11.04, SD = 1.16) assessed their life satisfaction, positive and negative affect in two study waves, while mothers and fathers assessed family resilience in the first wave. A dyadic data common fate model was employed to create latent variables representing family resilience. Three latent variables were: family problem-solving, family spirituality, and utilization of social and economic resources. Findings from the structural equation model indicated a positive association between higher levels of family problem-solving and increased children’s life satisfaction, alongside a negative relationship between higher family spirituality and negative affect. Parental assessments of social and economic resources utilization were not uniquely related to children’s life satisfaction, positive, or negative affect.

## 1. Introduction

According to the Theory of Change model [1], which is based on Bronfenbrenner’s theory of ecological systems [2], the well-being of families and parents is the basis for developmentally appropriate parenting and adequate child development. Family well-being is thus made up of various factors at the level of individuals and factors at the family level. Individual-level factors include the mental and physical health of parents, while family-level factors include, for example, family self-sufficiency or family resilience. In this research, we focus on family resilience as an important determinant of children’s well-being. Subjective well-being encompasses various cognitive and affective experiences, and it is usually viewed as a multidimensional construct. Cognitive aspects pertain to the overall appraisal of life or specific life domains, whereas affective components encompass individuals’ encounters with positive and negative emotions. [3]. Starting from the tripartite model of subjective well-being [4], we conceptualized that children’s subjective well-being consists of the three components of well-being. There is empirical evidence that the tripartite higher-order model (context-free life satisfaction, negative and positive affect) shows the best fit for the observed data on a sample of Croatian children and preadolescents [5]. In this study, the above-mentioned components are examined individually in order to examine how are the dimensions of family resilience related to each of the subjective well-being components.

The concept of psychological resilience was first explored in terms of an individual’s ability to adapt to adverse circumstances and was later applied to the level of family unit [6]. Family resilience refers to the family’s ability to strengthen family ties and enable personal growth for its members in conflict and stressful situations [1,7]. It can also be described as the capacity of a dynamic family system to resist or recover from significant challenges that threaten its stability, sustainability, or development [8]. Families with high resilience are characterized by clear and efficient communication between family members, a positive attitude, the ability to find meaning in adversity, spirituality, and the use of social and economic resources. Considering that children are a sensitive group that depends on the protection, support, and education of adults and that the family is one of the main sources of children’s socialization [9], it is worth exploring which family characteristics possibly predict children’s mental health and subjective well-being. Previous research indicated that different characteristics of parents and families predict different child outcomes [10], but relatively few studies focused specifically on the relationship between family resilience and children’s subjective well-being. Research on the relationship between family resilience and child’s subjective well-being that incorporates different data sources is also scarce.

Previous studies generally reported on a positive relationship between a family’s resilience and child’s subjective well-being. Masten [11] and Zolkoski [12] both highlighted the importance of family positive relationships, problem-solving skills, and areas of competence in children’s resilience. Hawley [13] and Patterson [14] further emphasized the role of family resilience in promoting positive outcomes for children, with the latter discussing the family’s subjective appraisal of stress and their ability to manage it. These findings collectively suggest that a resilient family environment can significantly contribute to a child’s subjective well-being. Previous studies also indicated a strong, positive connection between family communication and a child’s subjective well-being. Bireda [15] emphasized the criticality of transparent parent-child communication for the well-being of adolescents, while Schrodt [16] emphasized the importance of parental affirmation and affection regarding the child. Levin [17] underlined the important role of communication between both mothers and fathers with their children in children’s life satisfaction. The relevance of relationships with stepparents was noted as well. Knopp et al. [18] contributed by suggesting that fluctuations in parental communication patterns can lead to corresponding changes in child well-being over time. Taken together, these studies underscore a considerable effect of family communication on a child’s subjective well-being.

According to Walsh [19], family belief systems constitute the basis of family resilience, incorporating values, attitudes, and convictions that steer responses and behaviors amidst challenges. Walsh delineated three pivotal dimensions within family belief systems: the ability to find meaning in adversity, maintaining a positive outlook, and embracing spirituality or transcendence. Transcendental beliefs, frequently grounded in spirituality or religious principles, often offer clarity and comfort during turmoil, thus fostering favorable outcomes for individuals or families. Given the significance of spirituality, its role is often explored separately in existing research, acknowledging its impact on family functioning [20]. Pearce [21] also highlighted the enduring effects of family religious involvement on the quality of the mother-child relationship, which can in turn affect a child’s subjective well-being.

It is known from previous research that economic circumstances have a relatively small contribution to the explanation of children’s subjective well-being [22], especially when the circumstances are in the range of average, but only a smaller number of studies included the characteristics of the neighborhood in which the family lives as predictors of the child’s well-being. Some studies [23,24] showed that neighborhood quality (operationalized as neighborhood safety, availability of places to play, and social connectedness) predicts higher life satisfaction in children. Gross-Manos and Bradshaw [25] found that material deprivation is related with lower subjective well-being in children, while Main [22] highlighted the complex role of household income, which is mediated by factors such as material deprivation and perceptions of fairness. Phipps and Lethbridge [26] further supported this, demonstrating that higher family income is generally linked to better child well-being, particularly in cognitive and behavioral development. However, in other research [27] there was no relationship between neighborhood quality and children’s life satisfaction. This study aims to determine whether different components of family resilience, assessed by mothers and fathers, predict the child’s subjective well-being operationalized as cognitive (life satisfaction) and affective (positive and negative affect) well-being measured one year later.

## 2. Materials and Methods

This research was conducted as a part of the CHILD-WELL project, which is financed by the Croatian Science Foundation.

### 2.1. Participants

This study is based on a longitudinal project ‘Child well-being in family context’ (CHILD-WELL). Subsample for this study was chosen based on several criteria. Participants are included in the study if they had child-mother-father data in the first wave, if participants were not flagged for possible invalid responding, if at least two members of the triad indicated that the child lives with both parents in both waves and if they had data about sociodemographic variables (child gender and age, parent education and income). Based on these criteria, 762 child-mother-father (354 boys, age = 11.04, SD = 1.16) triads are included in the study. Mothers (age = 41.42, SD = 4.61) mostly had high school (45.4%) or college education (46.7%). Fathers (age = 43.90, SD = 5.26) also mostly reported high school (60.9%) or college education (32.9%).

### 2.2. Instruments

SLSS—Student Life Satisfaction Scale [28] evaluates children’s overall life satisfaction. Designed for individuals aged 8 to 18, this scale comprises 7 items assessing various cognitive assessments of life satisfaction. Participants assess their level of agreement with these statements using a Likert-type scale, ranging from 1 (strongly disagree) to 6 (strongly agree). The reliability of SLSS in both research waves is satisfactory (wave 1 α = 0.77, wave 2 α = 0.79).

PANAS-C—is an assessment tool used to measure both positive and negative affect in children and adolescents [29]. The shortened version of PANAS-C consists of two separate scales, one for positive affect (e.g., feeling ‘happy’, ‘excited’) and one for negative affect (e.g., feeling ‘upset’, ‘scared’). In total, it consists of 10 adjectives, five for each factor. Children rate the extent to which they experience the specified type of affect during the last few weeks on a scale from 1 (very little or not at all) to 5 (extremely). Children and adolescents are asked to rate the extent to which they have experienced each of the listed emotions during a specific time frame (e.g., the past few weeks) using a Likert-type scale. The reliability of PANAS-C subscales in both research waves is satisfactory (wave 1 PA α = 0.72, wave 2 PA α = 0.76, wave 1 NA α = 0.72, wave 2 NA α = 0.74).

FRAS—Family Resilience Assessment Scale is a questionnaire used to measure family resilience which refers to a family’s ability to withstand and bounce back from adversity or stress [30]. Shortened version of FRAS was assessed by both parents, measuring various dimensions of family resilience: family spirituality (4 items), maintaining a positive outlook (6 items), family connectedness (6 items), family communication and problem-solving (9 items), utilizing social and economic resources (8 items), and ability to make meaning of adversity (3 items). Mothers and fathers are asked to rate the extent to which they agree with the items using a Likert-type scale ranging from 1 (strongly disagree) to 4 (strongly agree). Subscales family communication and problem-solving, maintaining a positive outlook, and the ability to make meaning of adversity were highly intercorrelated for mothers and fathers (correlation range between 0.55 and 0.79). Because of that, these subscales are standardized and averaged for both parents to make one indicator of general family problem-solving. Factor family connectedness was excluded from further analysis due to low reliability (Cronbach α = 0.58 for both mothers and fathers). The reliability of FRAS subscales is high (Mother’s utilization of resources α = 0.87, father’s utilization of resources α = 0.86, mother’s spirituality α = 0.84, father’s spirituality α = 0.87, mother’s family problem-solving α = 93, father’s family problem-solving α = 0.94).

### 2.3. Procedure

Both children and their parents completed the questionnaire using the paper-pen method. Children completed their questionnaires at school, while parents completed theirs at home. A teacher or school psychologist was present in the classroom while the children completed the questionnaires. The first study wave was conducted in spring 2021 and second study wave one year later (spring 2022). By administering the questionnaires separately to children and parents, the study aimed to gather independent insights into family dynamics and individual perceptions.

## 3. Results

Before the main analysis, descriptive statistics and correlations among the study variables were examined. The descriptive parameters, as well as the reliability of each scale are shown in Table 1. Children rated their life satisfaction and positive affect highly at both time points, while the levels of negative affect are low. The reliability for each scale is satisfactory, and reliabilities of family resilience dimensions are somewhat higher than reliabilities of the children’s subjective well-being scales. In all structural equation models, we have used full information maximum likelihood method for treatment of missing data.

The correlations among the study variables are shown in Table 2. Regarding the subjective well-being measures in the first time point, life satisfaction has a positive, moderately high correlation with positive affect (r = 0.52, *p* < 0.001), and negative with negative affect (r = −0.39, *p* < 0.001). Positive affect is negatively related to negative affect (r = −0.29, *p* < 0.001). A very similar pattern of results emerged in the second time point, and correlations among subjective well-being measures are somewhat higher in the same time point (e.g., LS-NA, vs. LS-NA_2) which is expected. Regarding the correlations among the family resilience dimension, all intercorrelations are statistically significant, both within parent and between parents. The highest correlation is for the spirituality dimension (r = 0.72, *p* < 0.001). It is also worth noting that both the mother’s and father’s estimations of utilization of social and economic resources is not related to any of the child’s subjective well-being measures.

**Figure 1:**
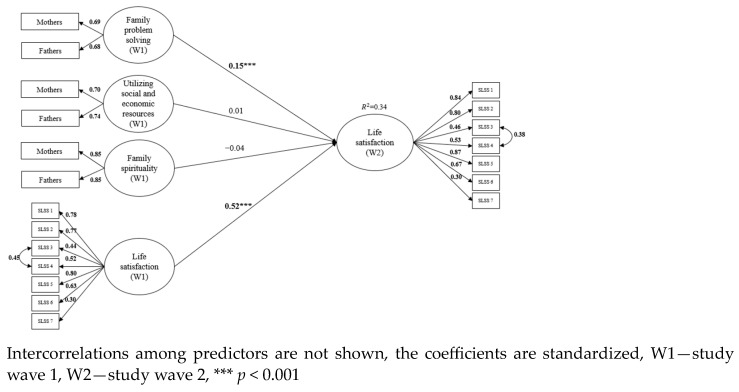
Family resilience dimensions (family problem solving, utilizing social and economic resources, family spirituality) as predictors of the child’s life satisfaction one year later.

**Figure 2:**
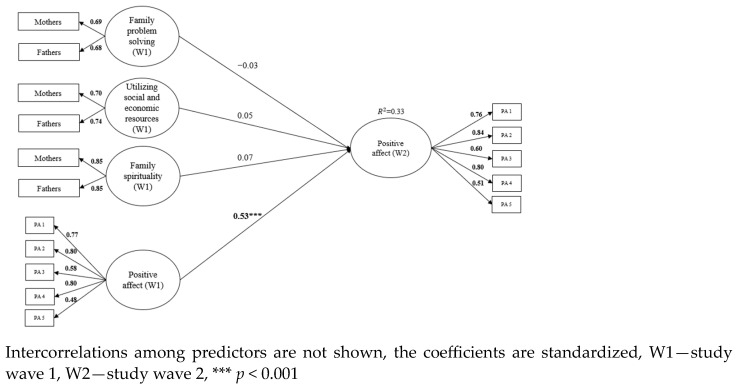
Family resilience dimensions (family problem solving, utilizing social and economic resources, family spirituality) as predictors of the child’s positive affect one year later.

**Figure 3:**
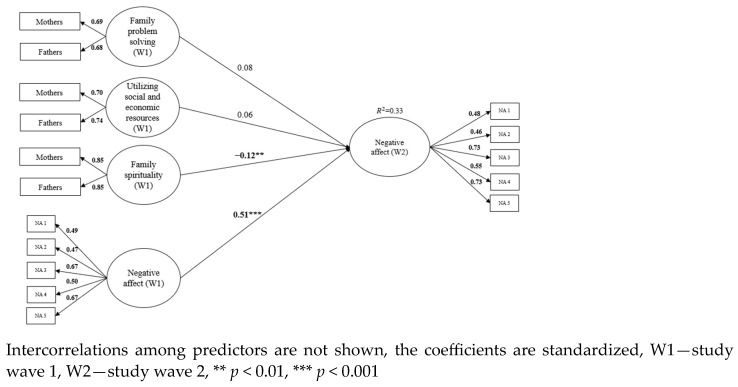
Family resilience dimensions (family problem solving, utilizing social and economic resources, family spirituality) as predictors of the child’s negative affect one year later.

Longitudinal invariance was examined for the child’s subjective well-being. All three measures of well-being demonstrated metric invariance which is sufficient for level of invariance for auto-regressive model. Family resilience was used as a predictor of changes in subjective well-being in three separate models. Both parents assessed all dimensions of family resilience, therefore a common fate model was used to form family level constructs for the three components of resilience (family problem-solving, family spirituality, utilizing social and economic resources). The common fate model for dyadic data posits that individuals within a dyad (pair) are influenced by shared environmental factors or experiences, leading to similar patterns of behavior or outcomes. In other words, they are interdependent and tend to co-vary over time [31]. Family level resilience factors were formed by fixing unstandardized loadings for both mothers’ and fathers’ reports at 1. In addition to the focal predictors, child age and gender, parent education, and family income (based on mothers’ reports) were included as control variables. The criteria variable from the first research wave was included as a predictor as well and shown in Figure 1, Figure 2 and Figure 3. Due to Chi-square sensitivity to sample size, models were evaluated based on several fit indices: Comparative Fit Index (CFI > 0.950), the Standardized Root Mean Residual (SRMR < 0.08), and Root Mean Squared Error of Approximation (RMSEA < 0.06) [32].

### 3.1. Model 1—Life Satisfaction

The first model consists of three predictors of the child’s life satisfaction—family problem solving, utilizing social and economic resources, and family spirituality. Mothers and fathers assessed all three predictors at first time point. Their estimations form a latent factor for each dimension. In addition to the focal predictors, child age, gender, parent education, family income (based on mothers’ reports), and criteria variable from first time point were included as control variables. The model shows good fit to the data (χ^2^ (249) = 392.009, *p* = 0.000, CFI = 0.970, RMSEA = 0.027, SRMR = 0.047). Family level problem-solving positively predicted increases in child’s life satisfaction a year later. In this model children’s age was a negative predictor of life satisfaction (β = −0.09, *p* < 0.01), and mothers’ education was a positive predictor of life satisfaction (β = 0.08, *p* < 0.05). The model is shown in Figure 1.

### 3.2. Model 2—Positive Affect

The second model consists of three predictors of the child’s positive affect—family problem solving, utilizing social and economic resources, and family spirituality. Mothers and fathers assessed all three predictors at the first time point and their estimations form a latent factor for each dimension. In addition to the focal predictors, child age, gender, parent education, family income (based on mothers’ reports) and criteria variable from first time point were included as control variables. The model shows good fit to the data (χ^2^ (165) = 258.116, *p* = 0.000, CFI = 0.976, RMSEA = 0.027, SRMR = 0.043). None of the dimensions of family resilience predicted changes in a child’s positive affect a year later. In this model, children’s age (β = −0.12, *p* < 0.01) and gender (β = −0.13, *p* < 0.001) were negative predictors of positive affect (female gender is related to lower levels of positive affect). The model is shown in Figure 2.

### 3.3. Model 3—Negative Affect

The third model consists of three predictors of the child’s negative affect—family problem-solving, utilizing social and economic resources, and family spirituality. Mothers and fathers assessed all three predictors at first time point. Their two estimations form a latent factor for each dimension. In addition to the focal predictors, child age, gender, parent education, family income (based on mothers’ reports) and criteria variable from first time point were included as control variables. The model shows good fit to the data (χ^2^ (163) = 239.740, *p* = 0.000, CFI = 0.974, RMSEA = 0.025, SRMR = 0.041). Only family spirituality predicted changes in negative affect a year later. Greater family spirituality predicted decreases in child’s negative affect. The model is shown in Figure 3. Children’s age (β = 0.20, *p* < 0.001), and gender (β = 0.13, *p* < 0.01) were positive predictors of negative affect (female gender is related to higher levels of negative affect).

In all three cases, predictors explain between 33–34% of the variance in criteria which is largely due to the strong contributions of criteria variables from the first study wave (β = 0.51–0.53, *p* < 0.01). To sum up, higher levels of family problem-solving predict higher levels of child’s life satisfaction a year later and family spirituality predicts lower levels of a child’s negative affect one year later.

## 4. Discussion

In order to gain more insight into the current state of the art in the field of children’s mental health and well-being, the relationship between family resilience assessed by fathers and mothers and children’s subjective well-being was investigated. Considering the lack of research on the resilience of the family as a system [33], this research provides insight into new knowledge about the connection between family well-being, i.e., family problem solving, utilizing social/economic resources, family spirituality, and the subjective well-being of children at the transition from middle childhood to adolescence. We explored children’s life satisfaction, and negative and positive affect as criterion variables. Overall, higher levels of family problem-solving (family communication and problem-solving, maintaining a positive outlook, and ability to make meaning of adversity), and family spirituality are related to higher levels of a child’s subjective well-being a year later. More precisely, higher levels of family problem-solving predict higher levels of the child’s life satisfaction a year later and higher levels of family spirituality are related to a child’s decreased negative affect a year later. Regarding the demographic variables, age was a negative predictor of life satisfaction and positive affect, and a positive predictor of negative affect. Mother’s education is positively related to the child’s life satisfaction, while female participants have higher levels of negative, and lower levels of positive affect. These results are largely in concordance with previous studies [5,34,35,36].

Family spirituality is negatively related to children’s negative affect. Families who go to church and are active in religious or spiritual activities have children who report less negative affect. That result is in concordance with most published research on this topic in which measures of family spirituality or religiosity are related to positive child-related outcomes [20,37,38]. The family assessment of the availability of social and economic resources did not predict the child’s subjective well-being The social and economic resources of the family in this conceptualization of family well-being mostly relate to the characteristics of the neighborhood in which the family lives and the availability of support from friends and neighbors. It includes statements like ‘we ask neighbors for help and assistance’ or ‘we feel people in this community are willing to help in an emergency’. In our research, economic and social resources were not associated with children’s well-being even at the bivariate level. Similar to other research [1,23,24], it is shown that indicators of the quality of family relationships, such as the quality of communication and problem-solving skills, are stronger predictors of the child’s well-being than distal predictors, such as the characteristics of the neighborhood in which the child grows up. Children in this study are aged between 9 and 13 years old, so they probably do not yet focus on broader social context and the position of their family in the community as much as adolescents and young adults would. Furthermore, utilization of family resources was assessed by parents, so it should be considered that the perspective of children possibly differs.

Family resilience research has encountered numerous criticisms, with significant attention directed towards issues of conceptual ambiguity and the term’s definition. The term ‘resilience’ has been subject to diverse interpretations, with researchers perceiving it as a trait, a process, and an outcome. A possible strength of this research is the conceptualization of family resilience itself. The three factors (family problem-solving, utilization of resources, and family spirituality) tap into different, but related dimensions of the concept. The intercorrelations among the resilience dimensions estimated by mothers range from 0.19 to 0.32 (*p* < 0.01) and fathers from 0.13 to 0.36 (*p* < 0.01) which supports the claim that this study covered different but connected dimensions of family resilience. Another strength is the fact that this study included three different data sources. In the study, children provided self-assessments of their subjective well-being, while parents offered assessments of family resilience. This methodological approach holds significant potential advantages, particularly in mitigating common method variance, as diverse data sources were utilized. In this case, children who tend to answer the questions with higher values or tend to view their surroundings more positively may give higher values on both resiliency and subjective well-being scales. Using different data sources, that problem is avoided. In addition to that, using both mothers’ and fathers’ estimations of family resilience increased the quality of the family resilience estimations. The results also show that mothers and fathers rate all three dimensions of family resilience highly and that associations between mothers’ and fathers’ ratings of the same dimension share from 22% to 52% of the family resilience variance.

### Study Limitations

There are several limitations to this study. First, the sample of families that participated in the CHILD-WELL project is tilted towards more functional and well-adapted families. Members of less functional families may hesitate to participate in a project focused on subjective well-being, parental behaviors, and family dynamics. Parents might have feelings of shame or inadequacy regarding these topics. More than 95% of the parents in the project are employed and have adequate financial resources, and children and parents rate themselves highly on subjective well-being scales. Furthermore, this subsample consists only of intact families where both parents live with their children. Those families are probably intact for a reason, meaning that, overall, it is logical to assume that the family dynamic is functional. In this sample, the parents rated their family’s resilience quite highly, which is shown in Table 1.

What might be of interest for researchers and practitioners is the reverse logic—is the absence of certain family resilience dimensions (e.g., economic resources) detrimental to the child’s well-being, and if so, which dimensions are the most important? To answer this question in future research, a different study design is required, and the study sample should be more representative of the general population. Lastly, the fact that this study is based fully on self-reported data is also a limitation, although self-reported data is usually a method of choice in this research field.

An auto-regressive structural equation model was used in this study because two data points were available for the analysis. Future researchers may consider incorporating more time points into their study to further understand the dynamic interplay between parental estimations of family resilience and children’s subjective well-being over time. By including multiple time points, researchers can capture the nuanced changes and trajectories in both constructs, providing a more comprehensive understanding of their relationship (e.g., latent growth model). These models allow for the examination of individual trajectories of change over time, capturing both within-person changes and between-person differences. By utilizing growth models, researchers can explore how parental estimations of family resilience predict changes in children’s subjective well-being over time, while also accounting for potential individual differences in these trajectories.

According to the Theory of Change model [1], different components of family well-being, family resilience being one of them, predict the child’s subjective well-being. According to this theory, economic and social resources are one of the family well-being indicators that should be positively linked to children’s well-being. That is not confirmed in this study since utilization of social and economic resources is not a significant predictor of any of the three well-being measures. The results of this study are only partially in concordance with the Theory of Change. The practical implications of this work tell us about the importance of a family’s problem-solving abilities, that is, family communication, maintaining a positive perspective, the ability to find meaning in adversity, as well as family spirituality, in contributing to the children’s subjective well-being. However, it also needs to be emphasized that dimensions of family resilience explain only a small percentage of variance in a child’s subjective well-being. Although family resilience plays a role in the child’s subjective well-being, other factors that are not accounted for in this research also determine subjective well-being. For example, various researchers established that certain biological and genetic factors partially determine the levels of an individual’s subjective well-being [39,40]. To sum up, strengthening family resilience may increase the mental health and subjective well-being of children and adolescents.

## 5. Conclusions

Results of this longitudinal study showed that higher levels of family problem-solving assessed by mothers and fathers were related to higher children’s life satisfaction in the second study wave, while higher family spirituality was related to lower negative affect in the second wave. Parental assessments of social and economic resources utilization were not uniquely related to children’s well-being, although the representativeness of the sample should be considered. Intact, functional families are overrepresented in this study and the study sample consists only of intact families (triads), respectively. The results are only partially in concordance with the Theory of Change and the dimensions of family resilience explained only a small percentage of variance in child’s subjective well-being.

## Figures and Tables

**Table 1:** Descriptive statistics for study variables.

	Min	Max	M	SD	α
LS (w1)	1.57	6	4.88	0.85	0.77
PA (w1)	1	5	4.19	0.69	0.72
NA (w1)	1	5	1.84	0.71	0.72
LS (w2)	1	6	4.93	0.79	0.79
PA (w2)	1.2	5	4.09	0.71	0.76
NA (w2)	1	4.8	1.89	0.70	0.74
M_res	1	4	2.88	0.57	0.87
M_spi	1	4	2.41	0.79	0.84
M_pro	1.7	4	3.50	0.37	0.93
F_res	1	4	2.86	0.54	0.86
F_spi	1	4	2.34	0.78	0.87
F_pro	1.44	4	3.48	0.40	0.94

Note. LS—life satisfaction; PA—positive affect; NA—negative affect; M—mothers, F—fathers; res—utilizing social and economic resources; spi—family spirituality; pro—family problem-solving; w1—first wave; w2—second wave.

**Table 2:** Correlations among the study variables.

	LS (w1)	PA (w1)	NA (w1)	LS (w2)	PA (w2)	NA (w2)	M_res	M_spi	M_ps	F_res	F_spi	F_pro
LS (w1)	1	0.52 ***	−0.39 ***	0.54 ***	0.28 ***	−0.32 ***	0.06	0.08 *	0.11 **	0.05	0.08 *	0.12 **
PA (w1)		1	−0.29 ***	0.42 ***	0.49 ***	−0.28 ***	0.04	0.09 **	0.14 **	0.04	0.12 **	0.14 ***
NA (w1)			1	−0.32 ***	−0.21 ***	0.44 ***	0.01	0.01	−0.09 *	0.03	−0.02	−0.10 **
LS (w2)				1	0.55 ***	−0.46 ***	0.04	0.04	0.15 ***	0.05	0.02	0.17 ***
PA (w2)					1	−0.43 ***	0.05	0.12 ***	0.08 *	0.07	0.09 *	0.06
NA (w2)						1	−0.07	−0.09 *	−0.06	−0.02	−0.08 *	−0.05
M_res							1	0.25 ***	0.32 ***	0.52 ***	0.17 ***	0.11 **
M_spi								1	0.19 ***	0.18 ***	0.72 ***	0.08 *
M_pro									1	0.18 ***	0.13 **	0.47 ***
F_res										1	0.30 **	0.36 ***
F_spi											1	0.13 ***
F_pro												1

*** *p* < 0.001, ** *p* < 0.01, * *p* < 0.05, M/F_res—utilization of family resources, M/F_spi—spirituality, M/F—problem solving; w1—first wave; w2—second wave.

## Data Availability

Data available on request due to privacy restrictions. The data presented in this study are available on request from the corresponding author.
